# Fungal keratitis caused by *Neurospora*: a case report

**DOI:** 10.3389/fmed.2024.1496010

**Published:** 2024-12-23

**Authors:** Yao Lu, Yijun Mo, Yuesong Weng, Xiaohui Li

**Affiliations:** ^1^Department of Laboratory Medicine, The First Affiliated Hospital of Ningbo University, Ningbo First Hospital, Ningbo, China; ^2^Department of Clinical Laboratory, The Affiliated Peoples' Hospital of Ningbo University, Ningbo, Zhejiang, China; ^3^Department of Ophthalmology, Ningbo Yinzhou No.2 Hospital, Ningbo Urology and Nephrology Hospital, Ningbo, Zhejiang, China

**Keywords:** *Neurospora*, fungal keratitis, keratitis, case report, corneal

## Abstract

**Background:**

We report a rare case of fungal keratitis caused by *Neurospora*, a filamentous fungus that is widely distributed in soil and graminaceous plants.

**Case presentation:**

A 40-year-old Mongoloid male patient came to our outpatient clinic with painful swelling of the left eye and redness, after being cut by a tree branch 1 week prior. After examination, the patient was diagnosed with a corneal ulcer of the left eye, and was given levofloxacin eye drops and levofloxacin ophthalmic gel. However, the patient did not respond to the treatment. After admission to the hospital, fungal mycelium was found in the corneal smear. To further identify the pathogen, a corneal scraping culture was used to extract fungal DNA and PCR amplification was performed using ITS universal primers, which was later sequenced and identified as *Neurospora*. We used fluconazole injections (0.2 g/100 mL) as eye drops to treat the patient once every hour, and itraconazole (200 mg) was administered orally once a day. After a few days, the patient’s condition improved.

**Conclusion:**

To the best of our knowledge, this is the first reported case of fungal keratitis caused by *Neurospora* in China. In this case, conventional topical and systemic treatment resulted in a favorable outcome. In patients with suspected fungal keratitis, medical treatment should be started urgently, and the treatment plan should be adjusted according to the subsequent experimental results and the patient’s condition.

## Introduction

Fungal keratitis is an infectious corneal disease caused by pathogenic fungi. Severe infection can lead to endophthalmitis or require corneal transplantation to restore vision. Although bacterial keratitis is more common than fungal keratitis, the latter have a poor prognosis, due to the lack of effective therapeutic drugs and methods of treatment, and also because fungi differ from other pathogens in their pathogenesis (1). At least 166 genera and 144 species of fungi have been reported to cause human fungal keratitis, including over 100 genera of filamentous fungi, 18 genera of yeasts or yeast-like fungi, and six genera of dimorphic fungi (2). The common pathogenic fungi causing keratitis are *Aspergillus*, *Fusarium*, *Candida*, *Curvularia*, and *Penicillium*, among which *Fusarium* and *Aspergillus* are the most common. The prevalence of fungal keratitis may vary greatly in terms of geographical location and climatic conditions. In China, the pathogens of fungal keratitis are mainly *Fusarium*, *Aspergillus*, and *Alternaria* (3, 4). Here, we report a case of *Neurospora* infection. *Neurospora* is the genus of a group of filamentous fungi, with *N. crassa* most often the best studied species, which has served as a model eukaryotic organism for nearly a century (5). To the best of our knowledge, there are eight previously published cases of *Neurospora* infection listed in PubMed. These case reports suggested that *Neurospora* primarily caused co-infections in immunocompromised patients with underlying diseases, or occupational asthma in healthy people. In China, there has been no report of fungal keratitis caused by *Neurospora* until the present report.

## Case presentation

**Figure 1:**
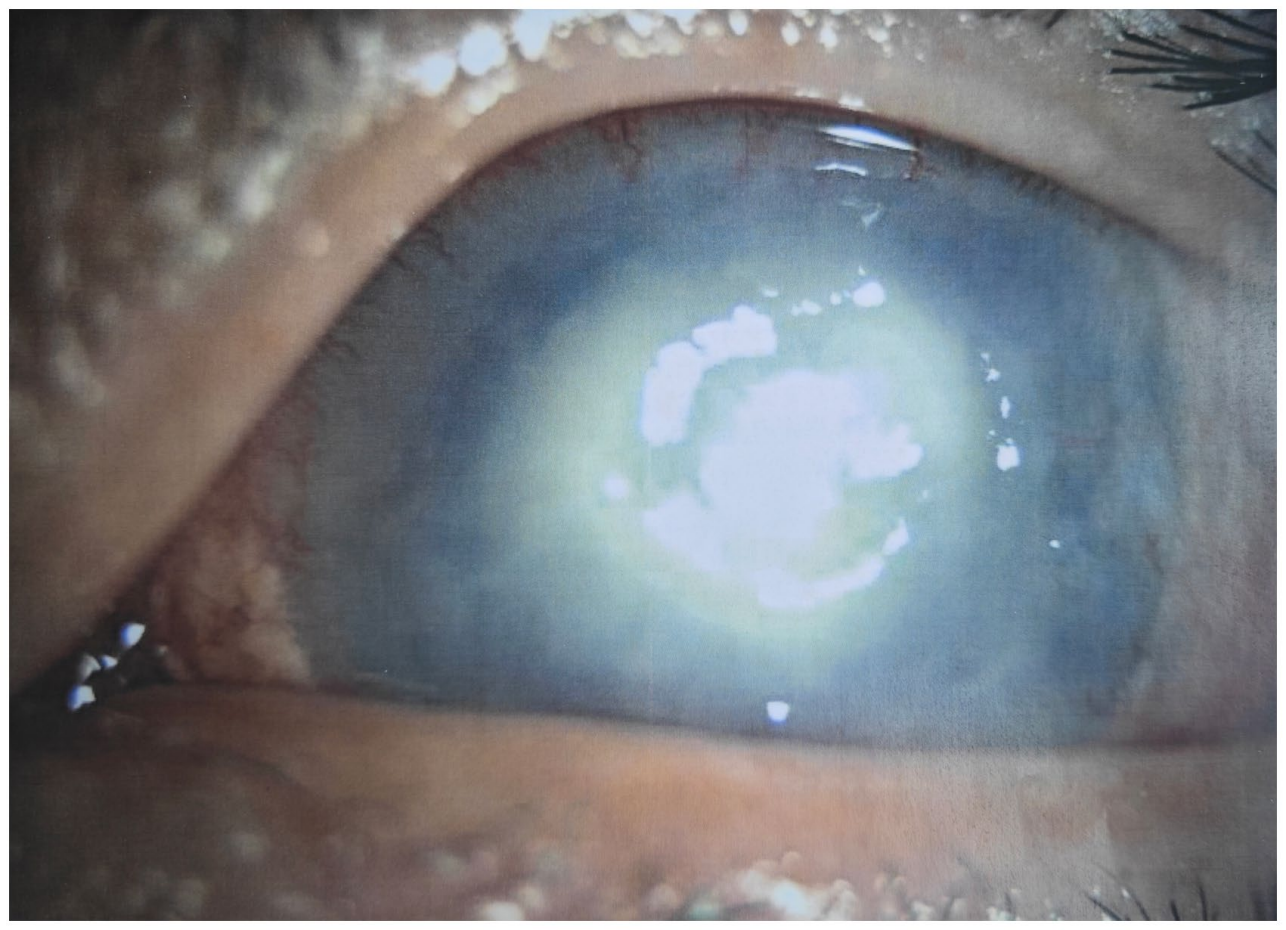
Slit-lamp photograph of left eye before treatment.

A 40-year-old Mongoloid male presented in our outpatient clinic with painful swelling of the left eye and redness, which was experienced for a week. One week earlier, after he was cut by a tree branch, the patient developed a swelling and pain in the left eye, accompanied by redness, but no obvious eye pain, photophobia, tearing, dizziness, headache, nausea, vomiting, or other discomfort. He was in good spirits, had a good appetite, slept well, had normal urine and stools, and had experienced no significant weight loss during his illness. The patient had type 2 diabetes mellitus for 5 years, was taking oral hypoglycemic drugs regularly, and claimed to have good blood glucose control. His visual acuity was 0.6, and his intraocular pressure was normal in the left eye. Slit-lamp examination revealed a cloudy, white, oval lesion with an indistinct margin and a dull surface on the temporal side of the left cornea. Pus was faintly visible in the anterior chamber below (Figure 1). A glaucoma drainage tube was observed at the 11 o’clock position in the anterior chamber, and the conjunctival filtering vesicles were flattened. Other details were unclear.

The patient was diagnosed with a corneal ulcer on the left side of the eye and was treated with levofloxacin eye drops and levofloxacin ophthalmic gel. However, he did not respond to this treatment. Therefore, we suspected that the patient had fungal keratitis, so he was admitted to our hospital for further treatment.

**Figure 2:**
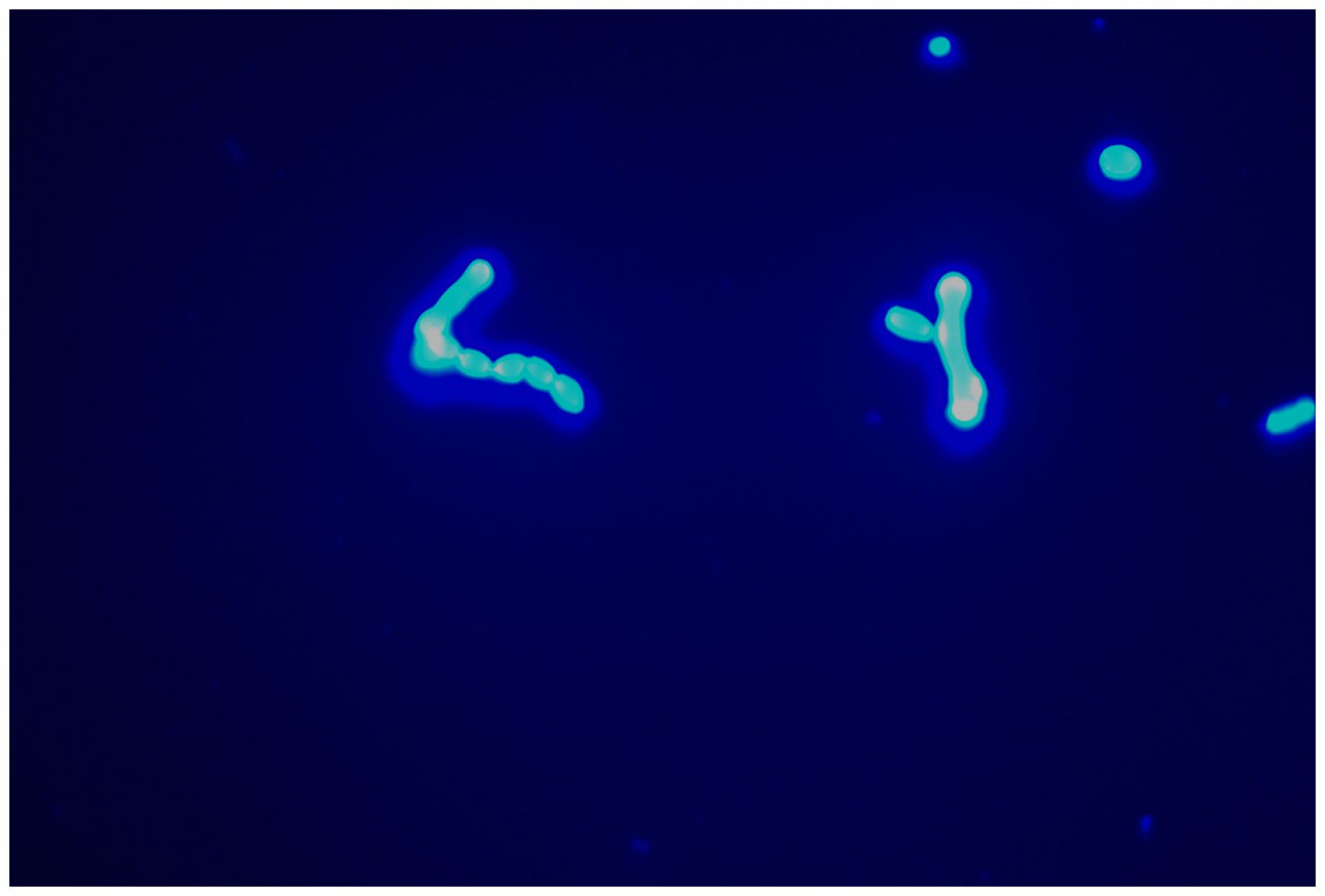
Mycelium could be seen with calcofluor white staining (original magnification, ×400).

**Figure 3:**
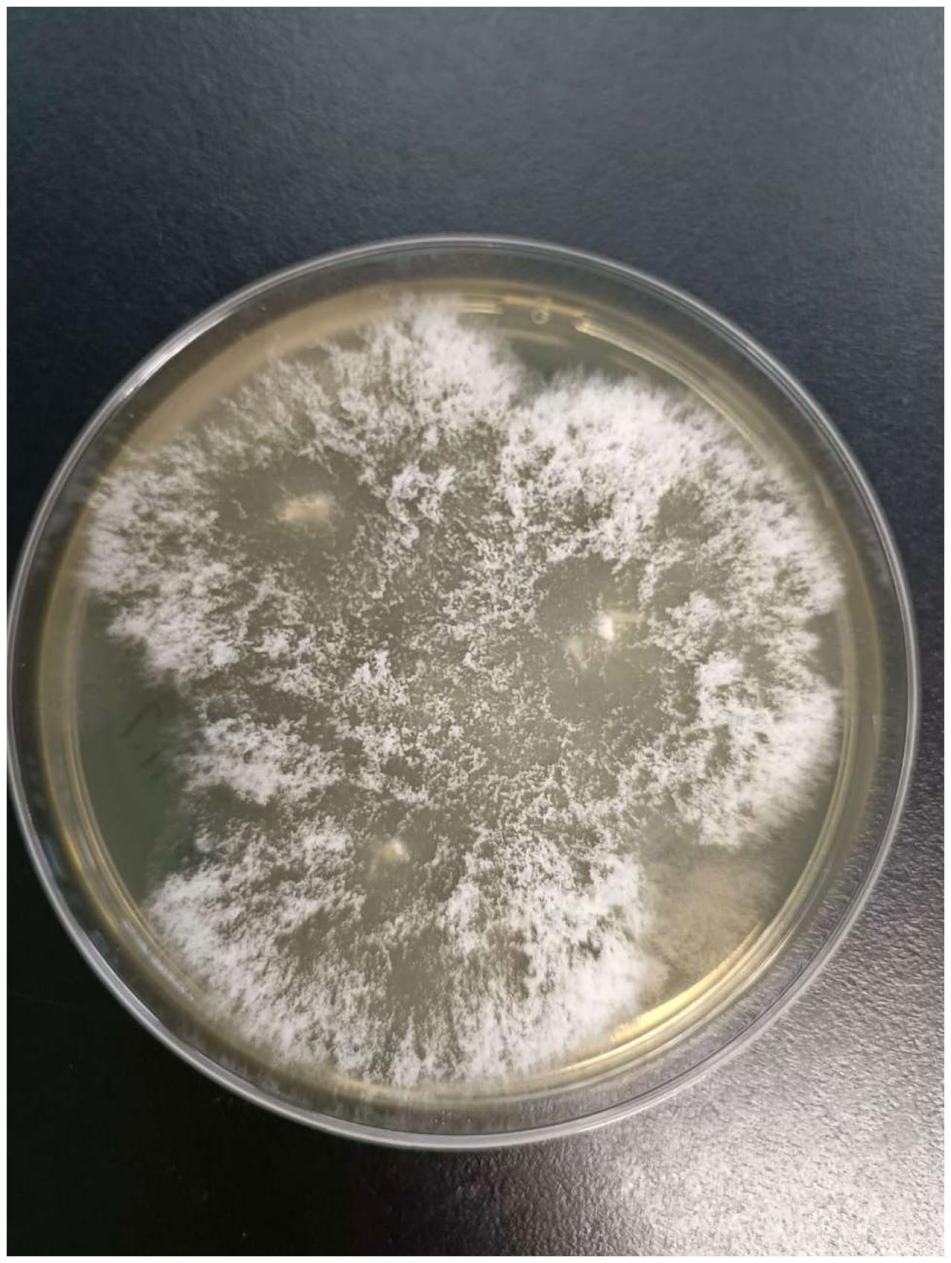
Colonies morphology of SDA medium cultured at 37℃ for 2 days.

**Figure 4:**
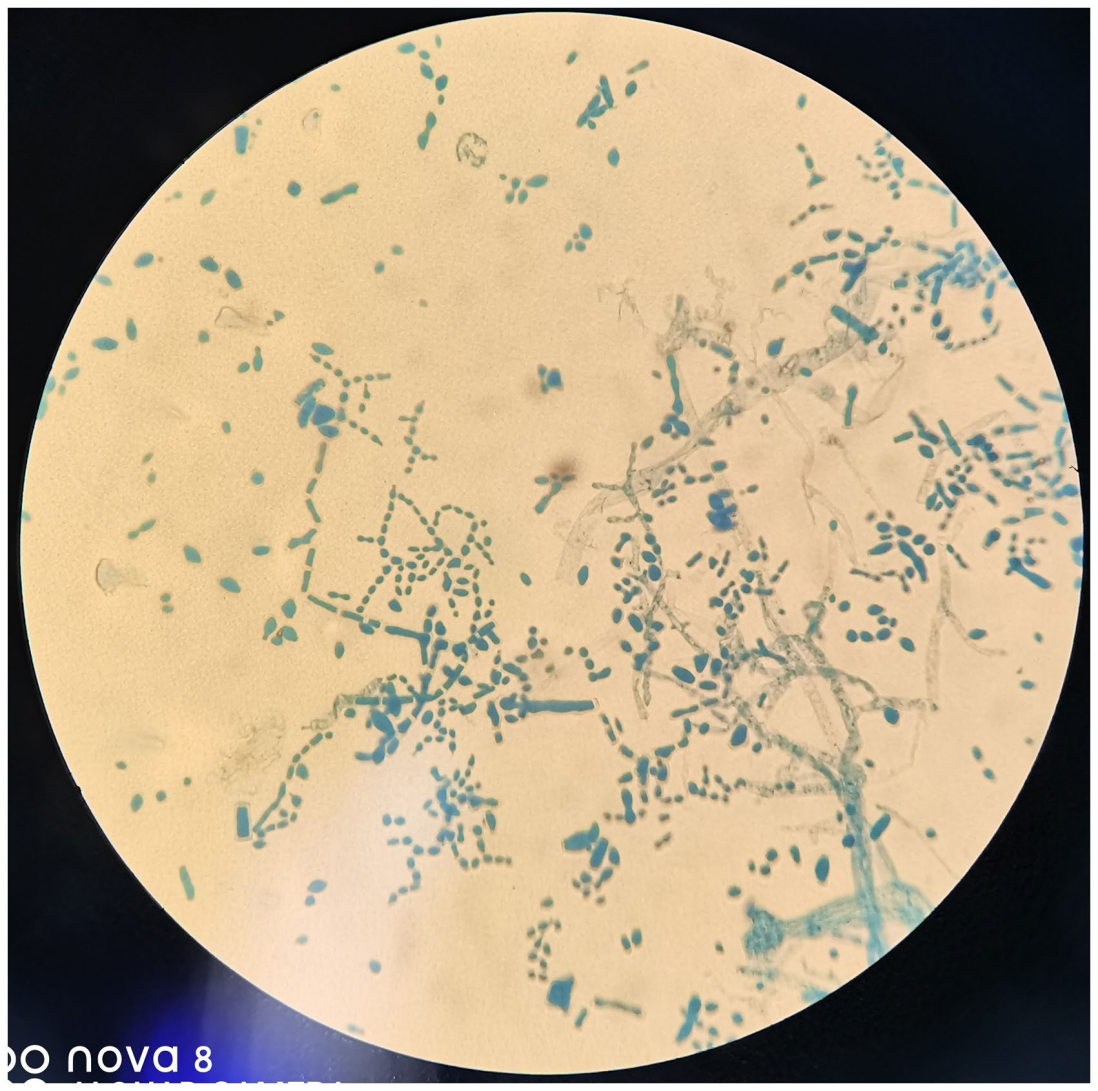
Columnar spore visible. Lactophenol cotton blue (original magnification, ×400).

After admission, the patient’s corneal surface was anesthetized and a specimen was scraped from the edge of the ulcerous corneal lesion. This was sent for microscopic evaluation and prolonged culture. A smear of corneal scrapings was stained for calcium fluorescence, and a few mycelium and columnar spores were seen using a microscope (Figure 2), while other scrapings were directly inoculated into Sabouraud glucose agar, and incubated at 37°C. After hyphae were detected, the patient was given topical fluconazole (FCZ) (0.2 g/100 mL) drops once every hour, and itraconazole (ICZ) (200 mg) was orally administered once a day. Meanwhile, we noticed a significant increase in his blood glucose, so we injected “Insulin Degludec and Insulin Aspart Injection” subcutaneously to control blood glucose levels. Two days later, small white colonies were cultured from his corneal scrapings by the hospital’s Mycology Department (Figures 3 and 4), apparently indicating a single fungus species. To exclude false positives due to contamination, repeated cultures from the edge of the ulcerous corneal lesion on a different occasion were conducted after mycelium were found in the direct smear of the corneal scraping, with the same white colonies being again cultured.

To further identify the pathogen, tissue cultures was sent for internal transcribed spacer (ITS) region amplification. The ITS region was amplified using primers: ITS1-F (5’-TCCGTAGGTGAACCTGCGG-3′) and ITS4-R (5’-TCCTCCGCTTATTGATATGC-3′). The cycling conditions were 95°C for 5 min, followed by 30 cycles of 94°C for 30 s, 57°C for 30 s, and 72°C for 90 s, and a final extension at 72°C for 10 min. Finally, it was identified as *Neurospora* using a DNA sequencing analysis of the internal transcribed spacer (ITS) gene at Sangon Biotech (Shanghai, China).

During the treatment period, the patient’s condition was stable, so we continued topical and systemic antifungal and glycemic control symptomatic supportive therapy. After treatment for 5 days, the patient’s corneal abscess regressed with minimal haze and his visual acuity was still 0.6. Therefore, he was discharged from the hospital and the antifungal medications were continued. During telephone follow-ups over the next 3 months, there was no sign of recurrence of keratitis.

## Discussion

Fungal infections rarely occur in healthy corneas, but plant trauma and contact lens usage are considered major risk factors. Over the past two decades, the increased incidence of fungal keratitis has been suggested to be related to the widespread abuse of broad-spectrum antibiotics, corticosteroids, and immunosuppressants (3, 6). *Neurospora* is an Ascomycete fungus and is widely distributed in soil and graminaceous plants. It was first found in 1843 as a contaminant in French bakeries, so it was also called “red-bread mold.” *Neurospora* is often considered a contaminant and commonly used in numerous eukaryotic cell biology studies (7). It has been implicated in peritonitis, eye infection, oral cavity infection, and occupational asthma (8). In patients with trauma or burns, prominent risk factors for *Neurospora* infection include incorrect steroid usage and diabetes (9). In the present case, the patient was cut by a tree branch and suffered from type 2 diabetes mellitus for 5 years. Despite the usual regular use of hypoglycemic drugs, a significant increase in blood glucose was found during hospitalization, which increased the likelihood of fungal infection with *Neurospora* species, which is normally considered non-pathogenic to humans. *Neurospora* is usually encountered as a contaminant, because of its rapidity of growth (10). In this case, repeated cultures from the edge of the ulcerous corneal lesion on two different occasions with concurrent symptoms made this highly improbable.

Early and rapid identification of pathogenic organisms is key to ensuring successful treatment of corneal infections, especially fungal infections of the cornea. The initial clinical diagnosis of fungal keratitis is usually based on major predisposing factors such as plant trauma, contact lens use, and prolonged use of topical/systemic antibiotics or corticosteroids, and the typical clinical manifestations are endothelial plaques, white infiltrates, hypopyon, satellite-like lesions, feathery margins, and corneal ulcers with toothpaste-like surfaces (11). Culture of corneal scrapes is the preferred initial test to identify the infecting organism. Polymerase chain reaction (PCR) tests and *in vivo* confocal microscopy (IVCM) can complement the diagnosis (12). Corneal smears and cultures are traditional tests used in the diagnosis of fungal keratitis. Fungal cultures are the gold standard, but they require long incubation times, usually at least 2–3 days, and speciation is sometimes difficult. Calcofluor White staining of corneal smears is a rapid, sensitive, and specific method for the diagnosis of fungal keratitis, but its sensitivity and specificity are still limited by the method itself. IVCM and PCR can improve diagnostic accuracy, but IVCM requires expensive equipment and trained, experienced operators to perform the technique. Regarding PCR, in addition to the limitations described above, contamination from the environment and commensals can result in a false positive result (13–15). For this rare fungal infection, traditional smears, as well as culture and morphology-based fungal identification, do not always provide enough resolution for identifying fungal species, and under these conditions, molecular detection would perform well in distinguishing *Neurospora* from other common keratitis-causing fungi.

There are many challenges in treating fungal keratitis. Two major problems in the treatment of fungal keratitis are the lack of laboratory diagnostic tools to determine drug sensitivity and poor ocular penetration, resulting in low drug bioavailability. Fungal keratitis is treated primarily with topical medications. For deep and refractory keratitis, intrastromal and intracameral injections are more effective. Amphotericin B (AMB), natamycin (NTM), and voriconazole (VCZ) are the only topical antifungal drugs currently used, with sufficient data indicating their efficacies, safety, and clinical indications (16). NTM is the usual first-line drug of choice for filamentous fungal keratitis. VCZ has also been reported to be effective against a broad spectrum of fungi, especially for non-Fusarium infections and when keratitis is caused by rare pathogens (17). For natamycin-resistant cases, AMB can be used to treat filamentous fungi, but penetration is poor and toxicity is high (18). In addition to topical medications, several clinical and experimental studies have reported favorable results with systemic azoles including ketoconazole, ICZ, and FCZ (19). However, there is no standard treatment protocol for *Neurospora*. Hood et al. (10) described a successful outcome with complete resolution of the infection in 4 months using FCZ (200 mg daily). In the present case, we used FCZ (0.2 g/100 mL) as eye drops once every hour and ICZ (200 mg daily) as a systemic antifungal to treat the patient. As a result, fungal keratitis caused by *Neurospora* was ameliorated.

## Conclusion

We reported the case of a patient who developed fungal keratitis after being cut by a tree branch. When the patient was diagnosed with fungal keratitis, we immediately started medical treatment. After *Neurospora* was identified, treatment of the patient, who responded to topical FCZ and systemic ICZ treatment, was continued, and the patient’s condition improved significantly after 5 days of treatment. We suggest that medical treatment should be started immediately in patients diagnosed with fungal keratitis, and the treatment plan should be adjusted according to the subsequent experimental results and the patient’s condition. We also aimed to increase awareness of medical workers to rare cases of fungal keratitis, and we have provided protocols for the diagnosis and treatment of fungal keratitis caused by *Neurospora*.

## Data Availability

The datasets presented in this study can be found in online repositories. The names of the repository/repositories and accession number(s) can be found in the article/supplementary material.
